# CT and MR Imaging Characteristics of Intravestibular and Cerebellopontine Angle Lipoma

**DOI:** 10.5812/iranjradiol.11320

**Published:** 2014-05-15

**Authors:** Ramazan Buyukkaya, Ayla Buyukkaya, Beyhan Ozturk, Huseyin Yaman, Abdullah Belada

**Affiliations:** 1 Department of Radiology, School of Medicine, Duzce University, Duzce, Turkey; 2 Department of Radiology, Duzce Ataturk Government Hospital, Duzce, Turkey; 3 Department of Ear Nose and Throat, School of Medicine, Duzce University, Duzce, Turkey

**Keywords:** Cerebellopontine Angle, Lipoma, Magnetic Resonance Imaging

## Abstract

Intracranial lipoma is an uncommon entity. A rare type of tumor in the internal auditory canal (IAC) and the cerebellopontine angle (CPA) is lipoma. There are a few case reports in the literature related to intravestibular lipoma. Herein, we report a case of lipomas within the cerebellopontine angle and vestibule of the inner ear in a patient with tinnitus and dizziness. The patient was evaluated with a 1.5 T magnetic resonance imaging (MRI) system. MRI and CT showed the masses in the left CPA and the left IAC. These lesions were hyperintense on both T1- and T2 weighted images and showed no enhancement after gadolinium administration. Conservative management was suggested. Histopathological diagnosis is rarely necessary with the widespread use of magnetic resonance imaging. Considering significant morbidity during resection, conservative follow-up is the best approach for CPA and IAC lipoma.

## 1. Introduction

Lipomas are asserted as ectopic fat that are formed secondary to lipomatous involution of the residue of meninx primitiva, the mesenchymal derivative of the embryonic neural crest, which envelops the developing embryo. Intracranial lipomas are most commonly observed at the midline, often with concomitant callosal or other midline anomalies, but can also occur in the suprasellar and pineal regions and rarely in the cerebellopontine angle (CPA) (1). Lipomas located in the internal acoustic canal (IAC) have been described (2). Herein, we report a case of synchronous lipomas in the CPA and the vestibule of the inner ear in a patient with tinnitus, dizziness and hearing loss.

## 2. Case Presentation

In 2011, a 26-year-old woman referred to our department complaining of a profound hearing loss in the left ear. Episodes of tinnitus and dizziness were reported in her history. Although the patient described dizziness, she did not have spontaneous nystagmus in physical examination and the Romberg test was negative, so advanced vestibular tests were not performed. Tonal audiometry revealed a 37-dB hearing loss in the left ear, with 70% speech discrimination. Right ear pure tone audiometry was within the normal range.

**Figure 1.**
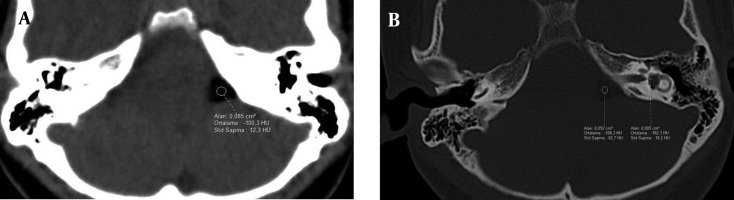
Axial CT images show lipoma as a hypodense lesion in A) the left cerebellopontine angle (HU of -100) and B) the left internal acoustic canal (HU of -182).

**Figure 2.**
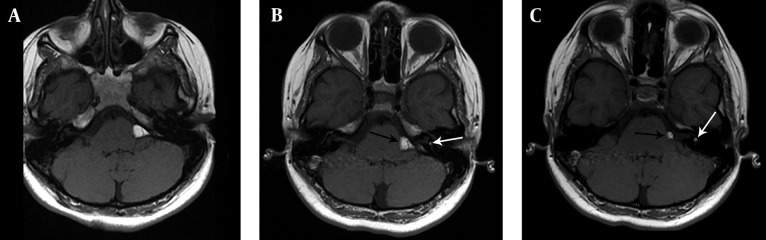
A, B and C) T1-weighted MRI images show lipoma presenting as hyperintense lesions in the left cerebellopontine angle (black arrows) and the left internal acoustic canal (white arrows).

**Figure 3.**
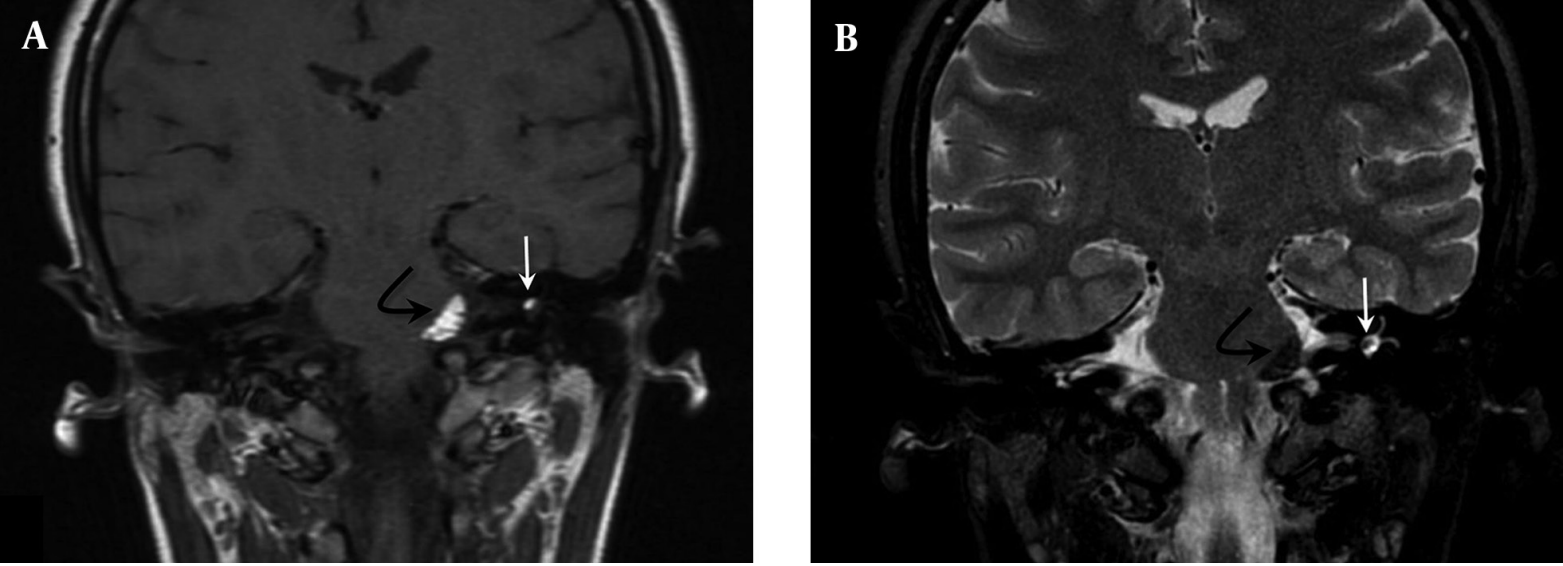
A) Coronal T1-weighted MRI image reveals hyperintense lesions in the left cerebellopontine angle (black arrow) and the left internal acoustic canal (white arrow); B) Coronal T2-weighted fat suppressed image reveals saturated hypointense lesions.

**Figure 4.**
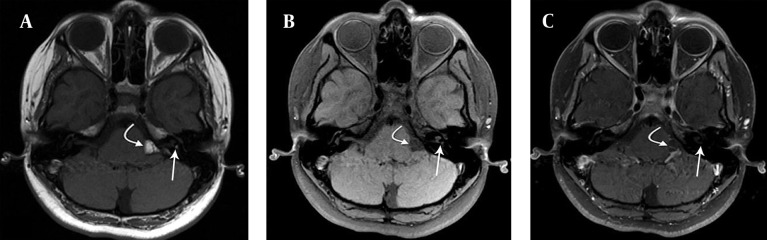
A) Axial T1-weighted image shows a hyperintense mass due to lipoma in the left cerebellopontine angle (curved white arrow) and left internal acoustic canal (straight white arrow). B) Axial T1-weighted image with fat saturation displays saturation of lipomas in the left cerebellopontine angle (curved white arrow) and left vestibule (straight white arrow). C) Enhanced axial fat saturation T1-weighted MR image shows no enhancement of lesions

The patient was subjected to radiological examination and computed tomography revealed a homogeneously hypoattenuated CPA mass (Figure 1). MRI showed a hyperintense 15×7 mm sized left CPA mass that encased the left VIII cranial nerve on T1-weighted images. The mass signals were suppressed on fat saturated images. The eighth cranial nerve was followed up and a 4 mm diameter lesion with a similar radiologic feature in IAC was seen (Figures 2 and 3). After administration of contrast with fat suppression, axial T1-weighted image displayed suppression of the CPA and intravestibular mass, and no enhancement was observed (Figure 4). These findings together with the confirmation on fat suppressed images were highly suggestive of a lipoma. Our patient was followed up clinically and radiologically for two years and there has been no progression.

## 3. Discussion

The largest proportion of CPA tumors are vestibular schwannomas and meningiomas, composing approximately 85-90% of the tumors seen in this location (3). Other lesions such as lipoma, papilloma, glioma or metastases account for less than 1% of CPA tumors in adults. Lipomas at this location are seen rarely, representing about 0.15% of the CPA lesions (4, 5). Very rarely, they are located in the IAC, and even less frequently, they have been described in an intravestibular location (6). Recently, Bigelow et al. (5) added 17 additional cases to 67 previously reported lipomas located in the CPA or IAC, but simultaneous occurrence of CPA and IAC lipomas are less frequent. We report a rare case of intravestibular lipoma concurrent with CPA lipoma.

Current theories on the pathogenesis consider lipomas as congenital malformations with one or two possible origins. A dysraphic disorder may cause mesodermal inclusions to remain trapped within the closing neural tube, or dysgenesis of indigenous tissue may cause the meninx primitiva to differentiate abnormally into adipose tissue (7, 8). Some authors have suggested that the mechanism of formation of intravestibular lipomas is similar or identical to CPA lipomas (9). As in our case, lesions that arise within or involve the IAC can be considered together with lesions affecting the CPA.

These tumors can cause symptoms related to the VIII nerve involvement, such as hearing loss, tinnitus, vertigo and nausea. Mukherjee et al. reported a study with 10 cases. Nine patients had hearing loss, five patients had unilateral and one patient had bilateral tinnitus, and two patients had vertigo (10). Our patient presented with unilateral tinnitus, dizziness and sensorineural hearing loss. Markou et al. studied seven female patients with a mean age of 51 years. Lesions were in IAC in four patients, and CPA in three patients. All were diagnosed with MRI and in all of them clinical and radiologic follow-up was recommended (11). Mukherjee et al. studied 10 patients of whom eight were male, the age range was between 22 and 71 years, six lesions were located solely within the IAC; two involved the CPA, whereas the remaining two had involvement of both regions. The average size of the lesions was 8 mm (range, 3-20 mm). One patient was operated because of progression of symptoms despite medical treatment.

Clinical and radiologic follow-up was recommended for the other patients (10).

White et al. studied 15 cases; eight lesions were confined to the IAC, while seven involved the CPA. The median tumor size at diagnosis was 7.2 mm. One patient underwent subtotal resection (12). Our patients’ lesion was 15×7 mm in size and was larger than the above mentioned series.

In these three series, the lesion was located in IAC in 18 out of 32 patients, in CPA in 12 out of 32 patients and in only two patients, the lesion was in CPA and IAC together similar to our case. Two of the 32 patients were operated and the others were followed up clinically and radiologically as in our cases. Due to their specific imaging findings, the diagnosis of intracranial lipomas is highly suggestive. The CT scan shows a marked hypodense nonenhancing lesion in the CPA, which has similar densities with adipose tissue (-40 to -100 HU). MR imaging demonstrates characteristic hyperintense lesions on T1-weighted MR and hypointense on T2-weighted MR images compared with the brain tissue. As in our case, they did not enhance after the administration of contrast agent. The use of MR imaging with fat suppression was extremely helpful to clearly demonstrate the lipomas.

As a result, lipomas must be kept in mind in the differential diagnosis. Careful radiologic evaluation is critical for correct diagnosis in order to prevent unnecessary intervention.
